# Cross-species complementation of bacterial- and eukaryotic-type cardiolipin synthases

**DOI:** 10.15698/mic2017.11.598

**Published:** 2017-11-03

**Authors:** Petra Gottier, Mauro Serricchio, Rita Vitale, Angela Corcelli, Peter Bütikofer

**Affiliations:** 1 Institute for Biochemistry and Molecular Medicine, University of Bern, Bern, Switzerland.; 2 School of Medicine: Basic Medical Sciences, Neuroscience and Sense Organs, University of Bari Aldo Moro, Bari, Italy.

## Abstract

The glycerophospholipid cardiolipin is a unique constituent of bacterial and mitochondrial membranes. It is involved in forming and stabilizing high molecular mass membrane protein complexes and in maintaining membrane architecture. Absence of cardiolipin leads to reduced efficiency of the electron transport chain, decreased membrane potential, and, ultimately, impaired respiratory metabolism. For the protozoan parasite *Trypanosoma brucei* cardiolipin synthesis is essential for survival, indicating that the enzymes involved in cardiolipin production represent potential drug targets. *T. brucei* cardiolipin synthase (TbCLS) is unique as it belongs to the family of phospholipases D (PLD), harboring a prokaryotic-type cardiolipin synthase (CLS) active site domain. In contrast, most other eukaryotic CLS, including the yeast ortholog ScCrd1, are members of the CDP-alcohol phosphatidyltransferase family. To study if these mechanistically distinct CLS enzymes are able to catalyze cardiolipin production in a cell that normally expresses a different type of CLS, we expressed TbCLS and ScCrd1 in CLS-deficient yeast and trypanosome strains, respectively. Our results show that TbCLS complemented cardiolipin production in *CRD1* knockout yeast and partly restored wild-type colony forming capability under stress conditions. Remarkably, CL remodeling appeared to be impaired in the transgenic construct, suggesting that CL production and remodeling are tightly coupled processes that may require a clustering of the involved proteins into specific CL-synthesizing domains. In contrast, no complementation was observed by heterologous expression of ScCrd1 in conditional TbCLS knockout trypanosomes, despite proper mitochondrial targeting of the protein.

## INTRODUCTION

**Figure 1:**
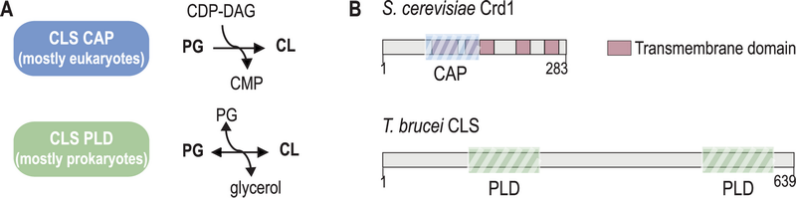
FIGURE 1: Types of cardiolipin (CL) synthases and their reaction mechanisms. **(A)** CLS belonging to the structural family of CDP-alcohol phosphatidyltransferases (CAP) catalyze an irreversible reaction between phosphatidyl glycerol (PG) and cytidine diphosphate diacylglycerol (CDP-DAG) to form CL and CMP, whereas CLS of the phospholipase D (PLD) type catalyzes a reversible transphosphatidylation reaction, using one molecule of PG together with another phosphatidyl donor (PG, PE, see main text) to form CL. **(B)** Schematic representation of the domain arrangement of CLS from yeast (*S. cerevisiae* Crd1; CAP-type) and *T. brucei* (TbCLS; PLD-type). The predicted transmembrane domains and conserved functional domains are indicated.

Cardiolipin (1,3-*bis*(*sn*-3’-phosphatidyl)*sn*-glycerol) (CL) is a unique glycerophospholipid of the energy transducing membranes of bacteria and mitochondria. Owing to its particular physicochemical properties, it plays an important role in the structural organization and the function of these membranes and strongly enhances the efficiency of the electron transport chain 1. Biosynthesis of CL occurs at the bacterial cell membrane and the inner mitochondrial membrane of eukaryotes, whereby two structurally and mechanistically distinct types of CL synthases (CLS) can be distinguished: eukaryotes typically express CLS enzymes belonging to the family of CDP-alcohol phosphatidyltransferases (CAP), which transfer the phosphatidyl moiety from CDP-diacylglycerol (CDP-DAG) to phosphatidylglycerol (PG) to synthesize CL. In contrast, most bacterial CLS use a second molecule of PG to form CL, catalyzed by a phospholipase D (PLD)-type transphosphatidylation mechanism (reviewed in 2). Exceptions from this classification are the CAP-type CLS of *Streptomyces coelicolor* and most likely other actinobacteria 3, and the PLD-type CLS of the unicellular eukaryote *Trypanosoma brucei* and all other trypanosomatids 4. In addition, putative PLD-type CLS were identified bioinformatically in the amoebozoan genus of *Dictyostelium* and in many genera of the group of alveolates, including *Plasmodium* and *Toxoplasma *5,6 (reviewed in 7). A systematic phylogenetic analysis of CL synthases led to the conclusion that CAP-type CLS in eukaryotes were most likely inherited from endosymbiotic alpha proteobacteria, the mitochondrial predecessors, whereas PLD-type CLS was already present in the first eukaryotic common ancestor 6. Subsequently, the more recent CAP-type CLS replaced PLD-CLS in the eukaryotic lineage from which multicellular eukaryotes emerged, while it got lost in the progenitors of the unicellular genera mentioned above 6. Figure 1 depicts the two different biosynthetic mechanisms of CAP- and PLD-type CL synthases and the domain organization of two representative members.

While it has been shown for several bacterial CLS, including *E. coli*, *Lactobacillus plantarum, *and* Bacillus ssp.,* that two molecules of PG are utilized to form CL (reviewed in 8), more recent studies have identified alternative substrates of PLD-type CLS. In a mass spectrometry-based *in vitro* activity assay of *E. coli* ClsC phosphatidylethanolamine (PE) was identified as the donor of the second phosphatidyl moiety 9. Interestingly, in addition to its CLS activity the paralog ClsB was found to also synthesize PG from PE and glycerol 10. In the plant pathogen *Xanthomonas campestris*, which contains six putative CLS genes, a PLD-type bifunctional PE/CL synthase was identified and proposed to synthesize PE from CDP-DAG and ethanolamine as well as CL from CDP-DAG and PG 11. The substrates of PLD-type TbCLS have not been identified.

The protozoan parasite *T. brucei* is endemic in sub-Saharan Africa where it causes sleeping sickness in humans and nagana in livestock. Unlike *E. coli* or *S. cerevisiae*, which are viable without a functional CLS, *T. brucei* fails to survive in absence of TbCLS in culture 4, making TbCLS an interesting drug target. In the context of a project to address the substrate utilization and mechanism of action of TbCLS, we studied whether PLD-type TbCLS and CAP-type *S. cerevisiae* CLS (ScCrd1) are able to functionally complement each other by introducing the genes into the respective (conditional) knock-out strains. Cross-species complementation of the two mechanistically different types of CLS has not been reported before.

## RESULTS AND DISCUSSION

### TbCLS functionally complements Crd1-deficient *Saccharomyces cerevisiae* (*crd1*Δ)

To assess the ability of TbCLS to complement ScCrd1-deficient *S. cerevisiae*, we used the commercially available *crd1*Δ strain Y03840, which is viable under standard growth conditions but exhibits a growth defect when cultured at elevated temperature or on non-fermentable media 12. To ensure correct and efficient mitochondrial targeting of TbCLS in *S. cerevisiae*, we fused the first 69 amino acids from the F_o_ ATPase subunit 9 from *Neurospora crassa* (in this study abbreviated with Su9) 13,14 to the N-terminus of TbCLS. Su9-*TbCLS* and a C-terminally tagged form (Su9-*TbCLS-HA*) were then expressed on an episomal plasmid in the *CRD1*-deleted strain (*crd1*Δ). As positive and negative controls for the phenotypic complementation analysis, *crd1*Δ cells were also transfected with the native *ScCRD1 *gene and the empty expression vector.

**Figure 2:**
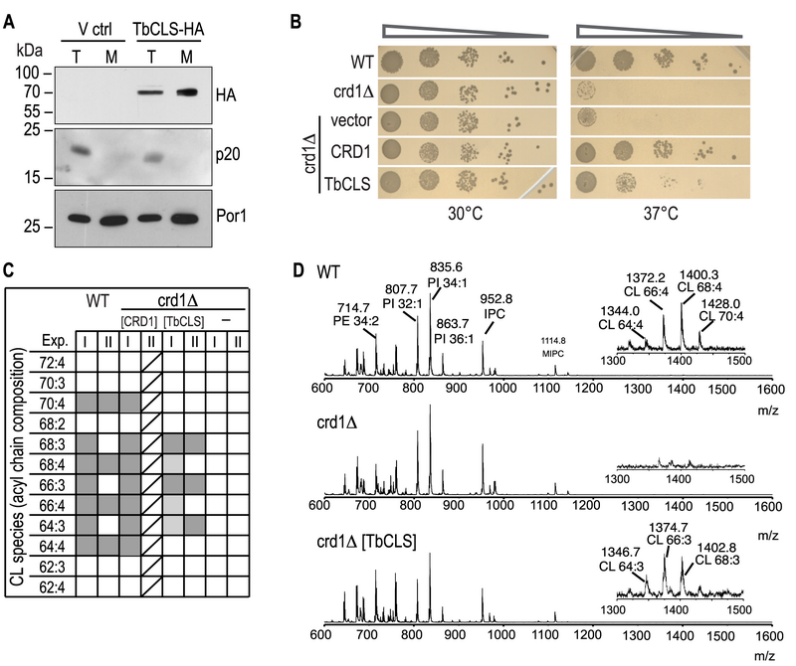
FIGURE 2: Expression, localization, and functionality of TbCLS in a *S. cerevisiae* *crd1*Δ background. **(A)** SDS-PAGE/immunoblot analysis using anti-HA antibodies of cell lysates (T) and mitochondria (M) prepared from *crd1*Δ cells transfected with the empty expression vector (V ctrl) or Su9-TbCLS-HA (TbCLS-HA). Antibodies against the cytosolic protein p20 and mitochondrial porin Por1 were used as controls. **(B)** Growth and colony formation in serial dilutions of *S. cerevisiae* wild-type (WT), *crd1*Δ, and *crd1*Δ cells expressing Su9-TbCLS, Crd1, or the empty expression vector. Plates were incubated in parallel at 30°C and 37°C for 4 days. **(C)** Cardiolipin (CL) species composition of wild-type (WT), *crd1*Δ (-), Crd1 addback (CRD1), and TbCLS complemented *crd1*Δ cells (TbCLS). Lipid extracts from crude mitochondrial membranes (Exp. I) or spheroplasts (Exp. II) were analyzed by MALDI-TOF mass spectrometry in two independent experiments. Molecular species are defined by the total number of carbon atoms in the acyl chains and the number of unsaturated bonds within the acyl chain. Identified species are marked in dark grey, minor species are in light gray; only one analysis was done with the CRD1 addback sample (indicated by diagonal lines). **(D)** Representative full-range mass spectra from experiment II; the insets highlight the m/z range of the cardiolipin molecular species of wild-type (WT, top panel) and TbCLS-complemented *crd1*Δ cells (crd1Δ [TbCLS], bottom panel); no cardiolipin species are detected in *crd1*Δ cells (middle panel).

To confirm expression and mitochondrial targeting of Su9-TbCLS, we analyzed protein extracts from whole cell lysates and isolated mitochondria from *crd1*Δ cells transfected with either the empty vector or Su9-TbCLS-HA by immunoblotting. As shown in Figure 2A, a specific band at about 70 kDa was detected in both total lysate and mitochondria-enriched fraction of Su9-TbCLS-HA expressing cells. The apparent molecular mass corresponds to the calculated molecular mass of TbCLS-HA of 74 kDa after cleavage of the Su9 targeting peptide.

To test functional complementation of *crd1*Δ by TbCLS, we first performed serial dilutions on SD-agar plates at 30°C and 37°C to compare growth and colony-forming capabilities of wild-type, *crd1*Δ*, crd1[CRD1] *and* crd1[Su9-TbCLS]* strains. As shown in Fig. 2B, at 30°C all transformants grew equally well. In contrast, yeast cells lacking ScCrd1 (*crd1*Δ) and mutants transfected with the empty vector were unable to grow at 37°C. Interestingly, expression of Su9-TbCLS in *crd1*Δ yeast was able to restore proliferation and colony formation on plates at 37°C at least partly. In contrast, despite proper expression and mitochondrial localization (see Fig. 2A), Su9-TbCLS-HA was unable to restore growth and colony formation of *crd1*Δ[Su9-TbCLS-HA] transformants at 37°C (data not shown), suggesting that the HA-tag interfered with the function of TbCLS.

To confirm CL synthesis by Su9-TbCls-expressing *crd1*Δ strains on a molecular level, we isolated lipids from spheroplasts and crude mitochondrial preparations and subjected them to MALDI-TOF MS (Matrix-Assisted Laser Desorption/Ionization-Time of Flight Mass Spectrometry) analysis. Figure 2C summarizes the observed CL molecular species distribution in extracts from wild-type, *crd1*Δ*, crd1*Δ*[CRD1], *and *crd1*Δ*[Su9-TbCLS] *strains from two independent experiments. Representative spectra from wild-type, *crd1*Δ*, *and *crd1*Δ*[Su9-TbCLS] *strains are shown in Fig. 2D. The results show that wild-type yeast produced the full spectrum of CL molecular species reported before 15,16,17, with the most abundant species having fatty acyl chains with a total of 64, 66, 68 and 70 carbon atoms and four unsaturated bonds per CL molecule (Fig. 2C, D). As expected, no CL molecular species were detected in the m/z range of 1200 - 1500 in *crd1*Δ cells (Fig. 2D). In contrast, in Su9-TbCls-expressing *crd1*Δ cells, the CL molecular species with fatty acyl chains with 64, 66, and 68 carbon atoms were present (Fig. 2D), demonstrating that CL synthesis was restored by Su9-TbCls in these cells. However, the masses of the CL molecular species differed by 2 dalton between wild-type and Su9-TbCls-expressing *crd1*Δ cells. In wild-type yeast, the most prominent species were at m/z 1372.2 (representing CL(66:4)) and 1400.3 (representing CL(68:4)) while in *crd1*Δ cells complemented with Su9-TbCls the major species were at m/z 1374.7 (representing CL(66:3)) and 1402.8 (representing CL(68:3)). The abundance of CL molecular species relative to other phospholipids was substantially lower in Su9-TbCls complemented cells compared to wild-type or *crd1*Δ*[CRD1]* cells, suggesting that the expression level or activity of Su9-TbCls was lower than that of ScCrd1.

The prevalence of triple-unsaturated CL species is reminiscent of the CL molecular species composition of the *cld1*Δ and, to some extent, *taz1*Δ mutants, in which remodeling of premature CL species is impaired due to the absence of the CL-specific deacylase ScCld1 17,18 or tafazzin 17,19. Deficient CL remodeling in Su9-TbCls-complemented cells may have different reasons: i) In wild-type yeast, ScCrd1 may be confined to specific domains within the inner mitochondrial membrane, possibly physically interacting with ScCld1 deacylase, or any other component of the remodeling machinery. In contrast, the heterologous enzyme TbCLS may not be directed to that specific microenvironment and a specific interaction with remodeling enzymes may be absent or deficient, resulting in the lack of CL remodeling. ii) ScCld1-mediated remodeling may only occur if CL levels reach a certain threshold. In Su9-TbCls-expressing cells, this threshold may not be reached.

### ScCrd1 does not complement a conditional *TbCLS* knockout cell line

In contrast to yeast, CL synthesis in trypanosomes is essential for cell proliferation and survival 4. To test whether ScCrd1 could functionally replace TbCLS, we expressed ScCrd1 in a conditional *TbCLS* knockout cell line of *T. brucei* procyclic forms, where the expression of the ectopic *TbCLS* recovery gene can be switched off by removing tetracycline from the growth medium 4. Using plasmids pGS-CRD1ΔLIIβ and pGS-CRD1-cMyc (details see experimental procedures), wild-type *ScCRD1 *or cMyc-tagged *ScCRD1* were stably integrated into the conditional *TbCLS* knockout genome along with the selectable marker gene *SAT1* (conferring nourseothricin resistance) and under the control of a constitutively active procyclin promoter. Transformed cells were selected in the presence of the antibiotic nourseothricin after limiting dilution in 24 well plates and genotyped by PCR.

**Figure 3:**
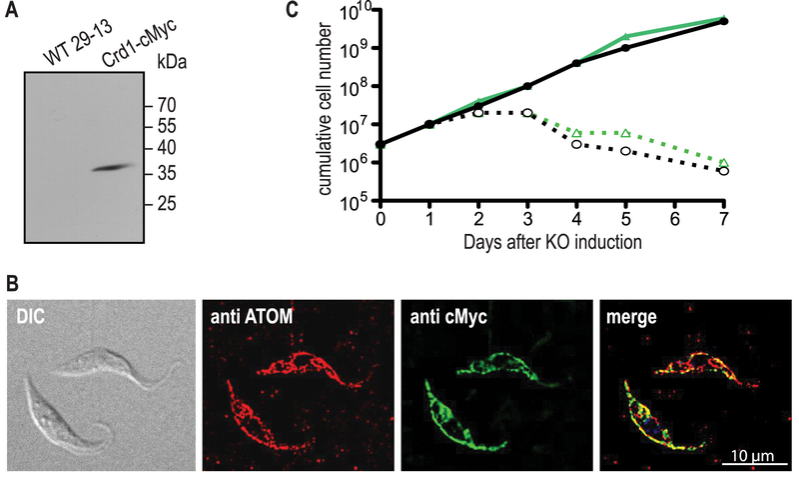
FIGURE 3: Expression, localization, and functionality of ScCrd1 in *T. brucei* *TbCLS* conditional knockout cells. **(A)** SDS-PAGE/immunoblot analysis of whole-cell extracts from wild-type (WT) and Crd1-cMyc-expressing parasites using anti-cMyc antibodies. **(B)** Immunofluorescence microscopy using antibodies against the endogenous mitochondrial outer membrane protein ATOM (red) and cMyc-tagged ScCrd1 (green). The overlay of both channels (merge) shows overlapping signals (yellow). DIC, Differential Interference Contrast. **(C)** growth curve of conditional *TbCLS* knockout cells expressing sccrd1. two different clones (black and green lines) were cultured in the presence (solid lines) or absence (dashed lines) of tetracycline (tet) to maintain or ablate, respectively, TbCLS expression.

Transcription of untagged ScCrd1 was examined by RT-PCR, confirming the presence of ScCrd1 mRNA. Expression of cMyc-tagged ScCrd1 on a protein level was confirmed by SDS-PAGE and immunoblotting of protein extracts from cells transformed with *ScCRD1-cMyc* (Fig. 3A). The subcellular localization of ScCrd1-cMyc in *T. brucei* was analyzed by immunofluorescence microscopy using antibodies against the cMyc epitope and against a protein of the archaic translocase of the outer mitochondrial membrane (ATOM) as mitochondrial marker 20. The results showed that the tagged protein is properly targeted to the mitochondrion, exhibiting the characteristic tubular staining throughout the cell (Fig. 3B).

To test viability of the transformants in absence of TbCLS, tetracycline was removed from the culture medium to ablate TbCLS expression 4. Growth curves of two clones expressing ScCrd1 grown in presence or absence of tetracycline were recorded and showed that ScCrd1 was unable to sustain parasite growth (Fig. 3C). Similar to *TbCLS* conditional knock out cells, proliferation in absence of tetracycline ceased after 48 h, followed by a continuous decrease in cell number and death of the culture. A similar observation was made for trypanosomes expressing cMyc-tagged ScCrd1 (not shown). Together, these data show that both untagged and tagged ScCrd1 are unable to complement growth of *T. brucei *procyclic forms after ablation of TbCLS, despite proper mitochondrial localization.

Several reasons for the inability of ScCrd1 to complement TbCLS in *T. brucei* are conceivable. Firstly, failure to cleave the mitochondrial targeting peptide in *T. brucei* may impair enzyme activity. As predicted by TargetP 1.1 21,22, ScCrd1 comprises a targeting signal with a cleavage site at Y53, resulting in a calculated molecular mass of 25.75 after proteolytic processing. Since the observed apparent molecular mass of cMyc-tagged ScCrd1 (Fig. 3A) likely corresponds to the molecular mass of the full-length protein (32.02 kDa) plus the C-terminal triple-cMyc tag (6.32 kDa), it is possible that ScCRD1 is not proteolytically processed in *T. brucei* parasites and may not be (fully) active.

Secondly, the finding that ScCrd1 in *S. cerevisiae* is embedded in a protein complex and that its activity is substantially reduced upon purification 23 suggests that ScCrd1 depends on the presence of other proteins for optimal function. These proteins may be missing in *T. brucei* or may not associate with ScCrd1. Conversely, since TbCLS is also part of a larger mitochondrial complex 4, it is possible that ScCrd1 is excluded from such a complex and, thus, from interacting with partners that are essential for CL formation in *T. brucei*.

Thirdly, Crd1 may not have access to its substrates, CDP-DAG and PG. It is known that the orientation of CLS differs between *E. coli* and mammals. While the active site of *E. coli* CLS is most likely directed towards the periplasm 24, CLS from rat liver mitochondria was shown to face the matrix side 25. Accordingly, being a homologue of bacterial CLS, the active site of TbCLS may be facing the intermembrane space while ScCrd1 may face the matrix. Thus, access of ScCrd1 to its substrates in *T. brucei* may be impaired. In addition, no mitochondrial CDP-DAG synthase has been identified in *T. brucei *26, implying that CDP-DAG as precursor for PG and CL synthesis would have to be transported from the endoplasmic reticulum to the inner mitochondrial membrane, a pathway that has not been demonstrated in *T. brucei*. Thus, it is possible that ScCRD1 may not be active since it has no access to its substrate CDP-DAG. Note that bacterial-type CLS do not depend on CDP-DAG as substrate for CL synthesis, i.e. a lack of mitochondrial CDP-DAG may not hamper CL formation by TbCLS in *T. brucei*.

### Summary

In this study we investigated the ability of two structurally non-related forms of eukaryotic CL synthases to functionally complement each other in cells lacking the native CLS. We found that PLD-type TbCLS is functional in yeast, alleviating the proliferation deficiency of *CRD1* knockout cells under stress conditions, whereas CAP-type ScCrd1 fails to substitute the essential function of TbCLS in trypanosomes.

We observed that CL produced by TbCLS in yeast contains less unsaturated fatty acids compared to natural mature CL of *S. cerevisiae*. A similar shift to a higher degree of saturation also occurs in CL from yeast strains with deficient CL-remodeling. The apparent lack of CL maturation in TbCLS-complemented *crd1*Δ cells suggests that biosynthesis and remodeling are tightly coupled processes, probably depending on a specific microenvironment and proper targeting of the involved proteins to specialized membrane domains. The non-homologous TbCLS may lack the property to localize correctly to such a microdomain or physically interact with the remodeling machinery.

## MATERIALS AND METHODS

### Reagents

All reagents were of analytical grade and, unless otherwise stated, purchased from Merck-Sigma Aldrich (Buchs, Switzerland). Kits for plasmid DNA extractions and PCR purification, Pfu polymerase and T4 DNA ligase were from Promega (Dübendorf, Switzerland). Restriction enzymes were purchased from ThermoFisher Scientific (Reinach, Switzerland). Home-made chemically competent *E. coli* XL-1 were used for molecular cloning. Growth media were prepared in the lab except for SDM79, for which a customized medium powder mix (Amimed^®^SDM79) was purchased from BioConcept (Allschwil, Switzerland).

### *T. brucei* strains, plasmids and growth media

The procyclic form *TbCLS* conditional knockout cell line containing a tetracycline-inducible copy of C-terminally hemagglutinin-tagged *TbCLS* has been described before 4.

For stable integration of *ScCRD1* or *ScCRD1-cMyc* into the *TbCLS* conditional knock-out cell line, plasmid pG-EGFPΔLIIβ 27 (kindly provided by Isabel Roditi, University of Bern, Bern, Switzerland) was used as backbone after replacing the neomycin (*NEO)* resistance marker by the nourseothricin resistance gene *SAT1*. pKOS 28 was taken as template for the amplification of *SAT1*. The triple-cMyc tag with flexible linker sequence to generate cMyc-tagged *CRD1* was PCR-amplified from the vector pJM1 (kindly provided by Jan Mani, University of Bern, Bern, Switzerland).

*TbCLS* conditional knockout parasites were cultured at 27°C in SDM79 supplemented with 15% (vol/vol) heat-inactivated FBS (Gibco^TM^, ThermoFisher Scientific, Reinach, Switzerland), 160 µM hemin, 90 µM folic acid, 15 µg/ml G418, 2 µg/ml puromycin, 5 µg/ml blasticidin, 0.2 µg/ml phleomycin, and 1 µg/ml tetracycline to induce the expression of ectopic *TbCLS. ScCRD1-*complemented *TbCLS* conditional knockout cells were cultured in the same medium with an additional 150 µg/ml nourseothricin (Jena Biosciences, Jena, Germany).

### Yeast strains, plasmids, and growth media

Designer deletion strain BY4741, referred to as wild type (WT) strain in this study, was used as control strain. The haploid *crd1*Δ (ORF YDL142c) strain Y03840 (BY4741*; MATa; ura3*Δ*0; leu2*Δ*0; his3*Δ*1; met15*Δ*0; *YDL142c*::kanMX4) *created in the course of the *Saccharomyces* Genome Deletion Project was purchased from Euroscarf and was used to test functional complementation with TbCLS.

The yeast episomal plasmid (YEp) pVT-H, a modified version of expression vector pVT-U 29 in which the *URA3* selection marker had been replaced by *HIS3*, was used for complementation of strain Y03840 with *TbCLS*. Another high copy number YEp, pDR197 (kindly provided by Doris Rentsch, University of Bern, Switzerland 30) was used for the addback expression of *ScCRD1* in strain Y03840.

Standard YPD growth medium YPD agar plates were used for general culture and maintenance of *S. cerevisiae* strains. For selections and large cultures of auxotrophic transformants, synthetic defined drop-out media (SD/DO) were used, composed of 0.67% yeast nitrogen base without amino acids, 2% dextrose, and the appropriate amino acids according to the Yeast Protocols Handbook (Clontech Laboratories) 31.

### ScCRD1 constructs for complementation of *T. brucei* CLS knock-out cells

Exchange of resistance marker of pG EGFPΔLIIβ 32: *SAT1* was amplified by PCR from plasmid pKOS using primer 1 and 2 (see table S1), digested with *Nhe*I and *Cla*I (*dam* sensitive) and ligated into the equally digested vector pG EGFPΔLIIβ isolated from *dam* *E. coli* strain GM48. The resulting vector was named pGS-EGFPΔLIIβ.

*ScCRD1* was amplified by PCR from isolated genomic DNA from strain BY4741 using primer 3 and 4 that were designed to replace *EGFP* from pGS-EGFPΔLIIβ by *CRD1* flanked by new multiple cloning sites.

For the construction of *CRD1-cMyc*, *CRD1* was first amplified using primer 5 and 6 from pGS-CRD1, digested with *Hind*III and *BamH*I and ligated into equally digested vector pJM1, fusing *CRD1* to a C-terminal triple-cMyc-tag. The fusion-sequence was subsequently PCR-amplified again using primer 5 and 7 followed by digestion with *Hind*III and *Pst*I. Ligation with *Hin*dIII/*Pst*I digested pGS-EGFPΔLIIβ resulted in pGS-CRD1-cMyc.

The resulting vectors pGS-CRD1 and pGS-CRD1-cMyc contained *CRD1*(-cMyc) and *SAT1* under the control of the constitutively active procyclin promoter and a modified procyclin 3’ UTR. Digestion with *Kpn*I and *Not*I allows genomic integration of the construct via homologous recombination with a procyclin locus.

### TbCLS and ScCRD1 constructs for complementation of yeast *crd1*Δ

ORF Tb427.4.2560, encoding *TbCLS*, was amplified from purified genomic DNA from *T. brucei* strain Lister 427 by PCR using primers 8 and 9 for the untagged copy and primer 10 for the construction of a C-terminally HA-tagged version. In both versions the thymidine base of the *BamH*I restriction site close to the end of the *TbCLS* ORF was mutated to cytidine.

The F_o_ ATPase subunit 9 from *Neurospora crassa*, in this study abbreviated with Su9, was PCR-amplified using primers 11 and 12 from template plasmid pGEM4 Su9(1-69)-DHFR 33. The fusion-sequence was obtained by sequential ligation of *Su9* and *TbCLS-HA* into the multiple cloning site of pDR197, resulting in the expression vector pDR-Su9TbCLS-HA.

For the expression of an addback copy of *ScCRD1* in *crd1*Δ, ORF YDL142c from *S. cerevisiae* was amplified by PCR from isolated genomic DNA from strain BY4741 using primers 13 and 14 and ligated into pDR197.

For the construction of untagged pVT H-Su9TbCLS constructs, the Su9 fusion sequences were PCR-amplified from pDR-Su9TbCLS-HA using primers 15 and 16, digested with *BamH*I and *Xba*I and ligated into equally digested pVT-H.

### Stable transformation of trypanosomes

Trypanosomes were grown to mid-log phase, harvested by centrifugation and resuspended in trypanosome transformation buffer 34 (90 mM sodium phosphate, 5 mM potassium chloride, 0.15 mM calcium chloride, 50 mM HEPES, pH 7.3) previously mixed with 10 µg of linearized DNA per 4 x 10^7^ cells. Electroporation was performed in 100 µl Nucleocuvettes™ using a Lonza 4D Nucleofector™ (X unit, pulse code FI-115, primary cells P3) as described in 35. Transformed cells were allowed to recover in standard growth medium without antibiotics during 24 h before limiting dilution and selective culture in presence of all previously used antibiotics and additional 150 µg/ml nourseothricin. 1 µg/ml tetracycline was added immediately after transformation and in all subsequent dilutions to keep the expression of ectopic TbCLS.

### Stable transformation of yeast

Transformation of yeast was performed according to the high efficiency transformation protocol by Gietz & Woods (2002) 36. Briefly, cells were grown to a density of approximately 2 x 10^7^ cells/ml and then washed in sterile water. Aliquots of 10^8^ cells were transferred to 1.5 ml tubes, pelleted and resuspended in 360 µl of transformation mixture containing 240 µl 50% PEG 3500 (w/v), 36 µl 1 M LiOAc, 50 µl 2.0 mg/ml single-stranded carrier DNA, 34 µl plasmid DNA (0.1 - 10 µg). Following incubation at 42°C for 40 min, cells were pelleted in a microcentrifuge, gently resuspended in 1 ml sterile water and plated onto selective medium.

### Determination of growth phenotype 

Functional complementation of the *crd1*Δ strain by TbCls was analyzed by making use of the temperature sensitive phenotype of the *crd1*Δ strain. Yeast WT, *crd1*Δ, *crd1*Δ[*CRD1*], *crd1*Δ[*Su9-TbCLS*], and *crd1*Δ [empty vector] strains were cultured in SD complete or the appropriate drop-out medium to an optical density (OD_600_) of 0.1, serially diluted by factors of ten and spotted on SD complete agar plates. Plates were incubated at 30°C or 37°C for 3 - 4 days and growth of the respective strains was compared between control (30°C) and stress (37°C) temperature.

### Phospholipid extraction and mass spectrometry 

Yeast spheroplasts and mitochondria-enriched fractions were prepared according to Gregg *et al.* 37. Generally, yeast cultures were grown to an optical density (OD_600_) of 0.8 - 2, washed 2 x with water and incubated in buffer containing 100 mM Tris/H_2_SO_4_ (pH 9.4) and 10 mM dithiothreitol [2 ml/g (wet weight) cells] for 20 min at 30°C with gentle shaking. Subsequently, cells were pelleted, washed, and resuspended in zymolyase buffer [20 mM potassium phosphate (pH 7.4), 1.2 M sorbitol] [7 ml of buffer/g (wet weight) cells]. 2 mg/g (wet weight) cells of zymolyase 20-T (AMS Biotechnology, UK) was added and the suspension was incubated with gentle shaking at 30°C until the OD_600_ reached 20% of the initial value (20 - 40 min). Isolation of crude mitochondria was done exactly following the protocol of Gregg *et al.* 37.

Lipids were extracted from washed spheroplasts (zymolyase buffer without zymolyase) or crude mitochondria using a modification of the protocol published by Bligh and Dyer 38: spheroplasts from up to 1 l of culture were resuspended in 8 ml 0.1 N HCl and transferred to a 250 ml Erlenmeyer flask containing 20 ml chloroform and 40 ml methanol. After incubation and shaking at room temperature for 1 hour, 20 ml chloroform, followed by 20 ml 0.1 N HCl were added under vigorous shaking. Phase separation was obtained after centrifugation at 2000 x *g *for 15 min. The organic phase was collected, dried under a stream of nitrogen and washed 1 x with a mixture of 0.1 N HCl:chloroform:methanol (0.8:2:2, by vol.). The washed and dried organic phase was dissolved in 1 ml chloroform: methanol (2:1, by vol.) and subjected to mass spectrometry.

MALDI-TOF MS was performed according to Angelini *et al.* (2012) 19 using a Bruker Microflex LRF instrument.

### Protein extraction, SDS-polyacrylamide gel electrophoresis and immunoblotting

Trypanosomes grown to a concentration of 10^7 ^cells/ml were harvested by centrifugation, washed with Tris-buffered saline (TBS; 10 mM Tris-HCl, pH 7.5, 144 mM NaCl) and resuspended in SoTE buffer (20 mM Tris-HCl, pH 7.5, 600 mM sorbitol, and 2 mM EDTA) to a concentration of 10^8^ cells/ml. Digitonin was added to the cell suspension at a final concentration of 0.25 mg/ml to lyse the cells. After incubation on ice for 5 min, non-solubilized membranes (including mitochondria) and cell debris were pelleted by centrifugation at 7000 x *g* in a table-top centrifuge, resuspended in sample buffer containing 2.5% SDS and boiled at 95°C for 5 min.

Yeast proteins from whole cells were extracted according to Kushnirov (2000) 39: Cells were pelleted, washed once with distilled water, and resuspended in 0.1 M NaOH (200 µl/2 mg of wet weight pellet). After incubation at room temperature for 5 min, cells were pelleted by centrifugation, resuspended in SDS-sample buffer, and heated at 50°C for 5 min. Proteins from yeast mitochondria were extracted by suspension of the mitochondria-enriched fraction in SDS-sample buffer and heating at 50°C for 5 min.

Proteins were separated on 12% polyacrylamide gels and transferred to polyvinylidene difluoride membranes (Millipore) by semi-dry blotting (Bio-Rad). Tagged proteins were visualized on films using SuperSignal West Pico (Pierce) chemiluminescent substrate after incubation with tag-specific primary antibodies and horseradish peroxidase-conjugated secondary antibodies. Antibodies used in this study were: mouse monoclonal antibody anti-cMyc (9E10; Santa Cruz Biotechnology, USA; dilution 1:1000 in TBS containing 5% skimmed milk); mouse monoclonal antibody anti-HA (HA11; Enzo Life Sciences, USA; dilution 1:3000 in TBS/5% milk); rat polyclonal anti-p20 antibody 40 (kindly provided by M. Altmann, Institute for Biochemistry and Molecular Medicine, University of Bern, Switzerland; dilution 1:1000 in TBS/5% milk); rabbit anti-porin antibody (kindly provided by R. Schneiter, Department of Biology, University of Fribourg, Switzerland; dilution 1:5000 in TBS/5% milk); rat anti-mouse HRP-conjugated secondary antibody (DAKO-Agilent, Switzerland; dilution 1:5000 in TBS/5% milk); goat anti-rat HRP-conjugated secondary antibody (Invitrogen; dilution 1:10000 in TBS/5% milk).

### Immunofluorescence microscopy of trypanosomes

Slides for immunofluorescence microscopy of trypanosomes were prepared as described in 41. Permeabilized and blocked [phosphate buffered saline (PBS) containing 5% skimmed milk powder] cells were incubated for 45 min with primary antibodies against cMyc (dilution 1:250 in blocking solution) and the mitochondrial outer membrane protein ATOM (archaic translocator of the outer membrane) (rabbit polyclonal anti-ATOM antibody, kindly provided by André Schneider, Department of Chemistry and Biochemistry, University of Bern, Switzerland; dilution 1:1000 in blocking solution). After washing, fluorescent secondary antibodies Alexa Fluor^®^ goat anti-mouse 488 and goat anti-rabbit 594 (Invitrogen™, diluted 1:1000 in blocking solution) were added for another 45 min. Following washing with PBS, slides were dried and coverslips were mounted with Vectashield^®^ containing 4’, 6-diamidino-2-phenylindole [DAPI (Vector Laboratories), Reactolabs, Servion, Switzerland].

Fluorescence microscopy was performed with a Leica DM 16000 B inverted microscope using a 60 x immersion objective and pictures were acquired using a connected Leica DFC360 FX camera. Image deconvolution and further processing was performed using Leica LAS X software and Fiji (NIH), respectively.

## SUPPLEMENTAL MATERIAL

Click here for supplemental data file.

All supplemental data for this article are also available online at http://microbialcell.com/researcharticles/cross-species-complementation-of-bacterial-and-eukaryotic-type-cardiolipin-synthases/.
