# Supplementary material for: Cross-species complementation of bacterial- and eukaryotic-type cardiolipin synthases
Source: Microb Cell. 2017 Nov 3;4(11):376–83. doi: 10.15698/mic2017.11.598 (PMC5695855; doi:10.15698/mic2017.11.598)
Supplement: Supplementary file 1 [file mic-04-376-s01.pdf]

SUPPLEMENTAL INFORMATION

TABLE S1 List of primers

Nr.	Name	Sequence 5`→ 3` (restriction sites underlined)
1	SAT1_N_f	TCTATTGCTAGCATGAAGATTTCGGTGATCCCTGA
2	SAT1_C_r	TTTAGTATCGATTGCATGCTTAGGCGTCATCCTGTGCTC
3	MCS_CRD1_f	TTTACTAAGCTTACCGGTGTCGACAGATCTATGATTCAAATGGTGCCCATTTATTCA
4	MCS_CRD1_r	TATACTGGATCCCTGCAGGAATTCCTCGAGCTATTTTAAAAGTTTAAAAGCGTTTCTCT
5	HindIII-AgeI-SalI-BglII fwd	TTTACTAAGCTTACCGGTGTCGACAG
6	Crd1 nostop_BamHI_rev	AGTATAGGATCCTTTTAAAAGTTTAAACGCGTTTCTCTT
7	PstI-Stop-cmyc rev	TGATTACTGCAGTCACAGGAGATCCTCCTCCGA
8	2560_SmaI_ATG_fwd	ACTACCCGGGATGACCTTCAATGGAAGGTTTCGTGCGAC
9	2560_BamHI_mut_rev	GTTAGGATCCTTAGTCTATCTCGGAGGAGAGATCGGGGTC
10	2560_HA_BamHI_mut_rev	GTTAGGATCCTTATGCATAGTCTGGTACGTCATAAGGGTAGTCTATCTCGGAGGAGAGATCGGGGTCC
11	Su9_XhoI_fwd	GTTACTCGAGATGGCCTCCACTCGTGTCCTCG
12	Su9_SmaI_rev	ACTACCCGGGGGAAGAGTAGGCGCGCTTCTG
13	Crd1_SmaI_fwd	ACTACCCGGGATGATTCAAATGGTGCCCATTTATTCA
14	Crd1_BamHI_rev	GTTAGGATCCCTATTTTAAAAGTTTAAAAGCGTTTCTCT
15	Su9_BamHI_fwd	CGCGGATCCATGGCCTCCACTCGTGTCCTCG
16	2560_XbaI_rev	CCCTCTAGACTAGTCTATCTCGGAGGAGAGATCGGG
